# Combined effects of the Pacific Decadal Oscillation and El Niño-Southern Oscillation on Global Land Dry–Wet Changes

**DOI:** 10.1038/srep06651

**Published:** 2014-10-17

**Authors:** Shanshan Wang, Jianping Huang, Yongli He, Yuping Guan

**Affiliations:** 1 Key Laboratory for Semi-Arid Climate Change of the Ministry of Education, College of Atmospheric Sciences, Lanzhou University, China; 2 State Key Laboratory of Tropical Oceanography, Chinese Academy of Sciences, South China Sea Institute of Oceanology, Guangzhou, China

## Abstract

The effects of natural variability, especially El Niño-Southern Oscillation (ENSO) effects, have been the focus of several recent studies on the change of drought patterns with climate change. The interannual relationship between ENSO and the global climate is not stationary and can be modulated by the Pacific Decadal Oscillation (PDO). However, the global land distribution of the dry–wet changes associated with the combination of ENSO and the PDO remains unclear. In the present study, this is investigated using a revised Palmer Drought Severity Index dataset (sc_PDSI_pm). We find that the effect of ENSO on dry–wet changes varies with the PDO phase. When in phase with the PDO, ENSO-induced dry–wet changes are magnified with respect to the canonical pattern. When out of phase, these dry–wet variations weaken or even disappear. This remarkable contrast in ENSO's influence between the two phases of the PDO highlights exciting new avenues for obtaining improved global climate predictions. In recent decades, the PDO has turned negative with more La Niña events, implying more rain and flooding over land. La Niña-induced wet areas become wetter and the dry areas become drier and smaller due to the effects of the cold PDO phase.

Drought is an extreme global climate event with major societal and economic consequences for millions of people in the world, especially in arid and semi-arid regions^1^,^2^. How patterns of drought are changing as the climate changes has been the focus of several recent studies, but the answer remains unclear.

Recently, Trenberth et al.^3^ demonstrated that natural variability, especially El Niño-Southern Oscillation (ENSO), is the primary cause of many episodic droughts around the world. Extensive research has documented ENSO-induced dry–wet anomalies over various regions^4^,^5^,^6^,^7^. However, the typical interannual relationship between ENSO and the global climate is not stationary and can be regulated by the Pacific Decadal Oscillation (PDO)^8^,^9^. Many studies have revealed that the PDO exerts a modulating effect on ENSO teleconnections over many parts of the world, such as the US^9^,^10^,^11^,^12^, South America^13^, Mexico^14^, Australia^15^, and East Asia^16^,^17^; however, the combined effect of ENSO and the PDO on global dry–wet changes remains unclear.

Here, we investigate the global-scale composite effect of different PDO phases on the ENSO-related dry–wet anomaly patterns, which may provide a better understanding of the ENSO's effects on dry–wet changes during the two phases of the PDO and can potentially improve forecasting of dry–wet changes. Because the influence of the tropical Pacific on climate is most pronounced in the boreal winter^18^,^19^,^20^, only December, January and February (DJF) are discussed herein.

## Results

**Figure 1:**
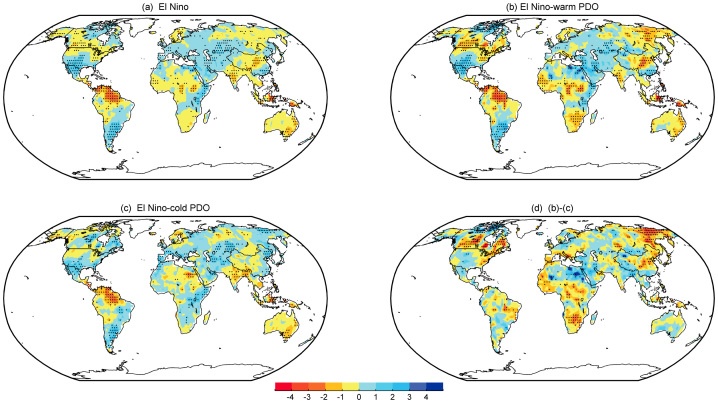
Composites of the DJF detrended sc_PDSI_pm for the period 1900–2012. (a) El Niño, (b) El Niño–warm PDO, and (c) El Niño–cold PDO. (d) Differences between in- and out-of-phase with the PDO, i.e., (b)–(c). The stippling indicates a 90% confidence level according to a two-tailed Student's t-test. Maps and plots were produced using licensed Matlab.

Figure 1 shows the winter mean sc_PDSI_pm for the El Niño composite and the sub-composites according to the PDO phase, together with the differences between in- and out-of-phase combinations, as indicated. The El Niño-related dry–wet changes result in drier conditions from southern and southeastern Asia to Australia (particularly along the eastern side of the country), central China, equatorial South America, the northern US, western and southern Canada, and the Sahel. Most other regions become wetter, particularly the central and southwestern US, northern Mexico, southern China, the southeastern coast of South America, the Horn of Africa, and the Mediterranean region toward central and southwestern Asia. Sub-composites with respect to the PDO phase reveal that the global El Niño-induced dry–wet changes are determined primarily by El Niño winters that occur during the warm phase of the PDO. In such winters (Fig. 1b), El Niño produces a similar (correlation coefficient of 0.83) but stronger pattern compared to that depicted in Fig. 1a. Significant drying over southern Canada, the Sahara to the Gulf of Guinea, southern Africa, central China and eastern Siberia, and wetter conditions over the Middle East, central Asia, the southern US and northern Mexico are more prominent and intense. In contrast, El Niño winters that occur during the cold phase of the PDO are generally similar to that depicted in Fig. 1a (correlation coefficient of 0.64), especially over Indonesia, Australia, southern China, the southeastern US, the southwestern US, northern Mexico, South America, and the Horn of Africa; however, the anomalies are weaker and exhibit some regional differences (Fig. 1c). For example, there is nearly no dry–wet change over the Sahel and western Asia and central China. Minor exceptions exist over southern Africa and eastern Siberia. In these regions, the changes reverse (i.e., get wetter) relative to Figs. 1a and 1b. Overall, the typical El Niño-induced dry–wet changes weaken and the drier area contracts during the cold PDO phase. The El Niño signal in the dry–wet distribution exhibits notable differences between the in- and out-of-phase cases (Fig. 1d). Clearly, El Niño induces more severe droughts in the warm PDO phase than in the cold phase over northern North America, northern South America, especially Brazil, sub-Saharan Africa, the Mediterranean basin, northeastern Asia and eastern Siberia, while it induces wetter conditions over southwestern North America, Uruguay, the Middle East, and southern China. Moreover, the droughts over the tropical western Pacific region that are associated with El Niño during the two PDO regimes are different; the drought is stronger from Indonesia to northeastern Australia during the warm phase of the PDO.

**Figure 2:**
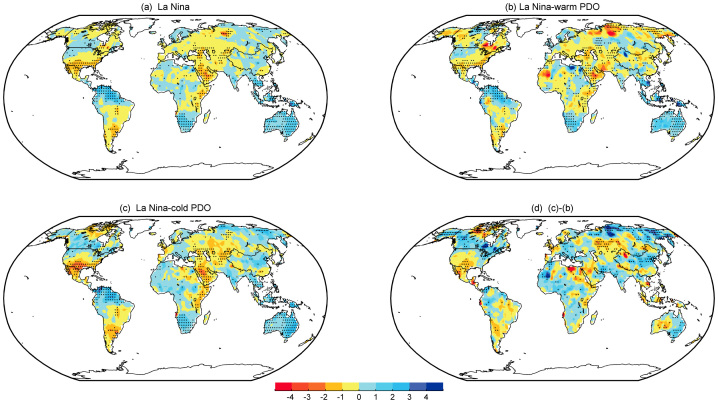
Same as in Fig. 1, but for La Niña. Maps and plots were produced using licensed Matlab.

Figure 2 is same as Figure 1 except for La Niña. The result shows that the La Niña related sc_PDSI_pm patterns are largely the inverse of the El Niño patterns (cf. Figs. 1a and 2a). However, the inverse signal is stronger over central and western Asia, the southeastern US, Australia, and southern Africa but weaker over the Sahel, central China, and southern Europe. Another minor discrepancy is that the northwest–southeast gradient separating the wet northwestern US and southwestern Canada from the dry eastern and southern US and northern Mexico is different from the north–south gradient depicted in Fig. 1a. The effects of the two PDO phases exhibit a rather weak response and small spatially incoherent signals during the out-of-phase (warm) condition (Fig. 2b), and with accentuated La Niña signals during the in-phase (cold) condition (Fig. 2c). As presented in Fig. 2b (Fig. 2c), the correlation coefficient between the canonical La Niña pattern shown in Fig. 2a and sub-composite La Niña anomalies during the warm (cold) phase of the PDO is 0.68 (0.76). The main difference in La Niña-related dry–wet changes between in- and out-of-phase conditions is that more precipitation occurs with greater intensity and a smaller region is subject to relatively severe drought during the in-phase conditions (Fig. 2d). A similar conclusion applies in summer that ENSO-induced dry–wet changes are magnified when in phase with the PDO with respect to the canonical pattern; however, the amplitudes are reduced compared to that in winter (Supplementary Figs. S1 and S2).

**Figure 3:**
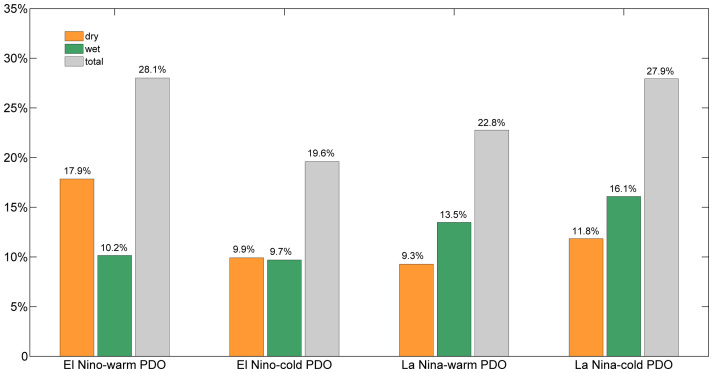
Fractions of the global land area with significant drying and wetting induced by ENSO during warm and cold PDO regimes. The significant drying and wetting can be observed in the stippling in Figs. 1 and 2, which is greater the 90% confidence level according to a two-tailed Student's t-test. Maps and plots were produced using licensed Matlab.

Figure 3 shows the fractions of global land area with significant dryness and wetness induced by ENSO in the two different PDO phases, which is indicated by the stippling in Figs. 1 and 2. The El Niño-induced drying when in phase with the PDO covers approximately 18% of the global land area, which is nearly double the coverage during the out-of-phase condition. The intensity of the El Niño-induced wetness is much more severe compared to the induced changes in the cold phase (Fig. 1d); however, the affected area is insignificantly larger. When La Niña occurs during the cold PDO phase, approximately 16% (12%) of the global land area suffers abnormal wetness or flooding (significant dryness or drought), which is approximately 20% (27%) more than that in the warm PDO phase. Overall, when in phase with the PDO, the ENSO-induced dry–wet changes are magnified; approximately 28% of the global land area is affected by the significantly abnormal dryness and wetness.

Composites of the detrended Climatic Research Unit (CRU) precipitation anomalies and the Global Land Data Assimilation System (GLDAS) surface soil moisture (0 ~ 10 cm) are also examined (Supplementary Figs. S3 and S4). The composites are roughly consistent with the sc_PDSI_pm changes; however, small regional differences exist. The small regionl differences between the sc_PDSI_pm and the precipitation are because the sc_PDSI_pm represents a cumulative departure in surface water balance^7^,^21^, determined not only by the current precipitation changes but also influenced by the evaporation and the preceding precipitation anomaly. Comparing ENSO-induced precipitation anomalies in combination with the two PDO phases demonstrates that the El Niño (La Niña) signal tends to be stronger during the warm (cold) phase of the PDO, which is similar to the findings for the sc_PDSI_pm. In addition, the coherence of the sc_PDSI_pm and precipitation is relatively high during the in-phase condition and is slightly different in a few regions during the out-of-phase condition of the ENSO and the PDO. For the soil moisture, the differences primarily occur over the northern high latitudes because the GLDAS dataset does not adequately represent snow and other processes that affect soil moisture^22^.

Similar changes in the sc_PDSI_pm are also observed in the dry–wet variations since 1950 when instrumental records have been relatively abundant (Supplementary Fig. S5). Furthermore, to verify the repeatability from one winter to another, the stability is assessed via the cross-validated proportion of the intra-composite variance (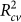) explained by the composite mean (Supplementary Fig. S6; see Method section for details). The stability of the sc_PDSI_pm signals reveals that the strong and significant dry–wet changes described above are typically stable and consistent from one winter to another. When ENSO and the PDO are in phase, the stable ENSO signals in the sc_PDSI_pm intensify and expand over the southern US, equatorial South America, southern Africa, central and western Asia, eastern Siberia, and from Indonesia to northern and eastern Australia.

**Figure 4:**
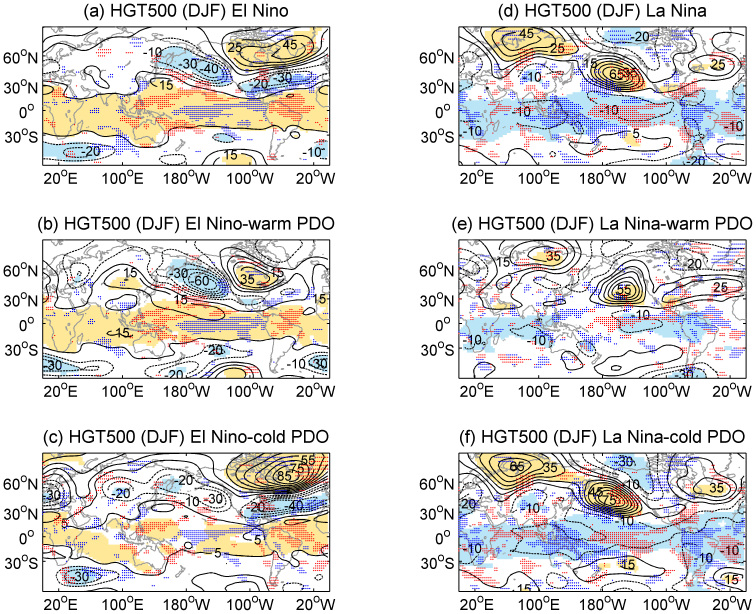
Composites of the DJF detrended 500 hPa HGT anomalies (contours, gpm, interval = 10 ghm) and winter vertical wind omega anomalies (the stippling, pascal/s) for the period 1950–2012. (a) El Niño, (b) El Niño–warm PDO, (c) El Niño–cold PDO, (d) La Niña, (e) La Niña–warm PDO, and (f) La Niña–cold PDO. Positive (negative) values are denoted as solid (dashed) lines with suppressed zero values. The areas with color shading indicate a 90% confidence level. The blue (red) stippling indicates negative (positive) omega anomalies greater than 90% confidence level. Maps and plots were produced using licensed Matlab.

**Figure 5:**
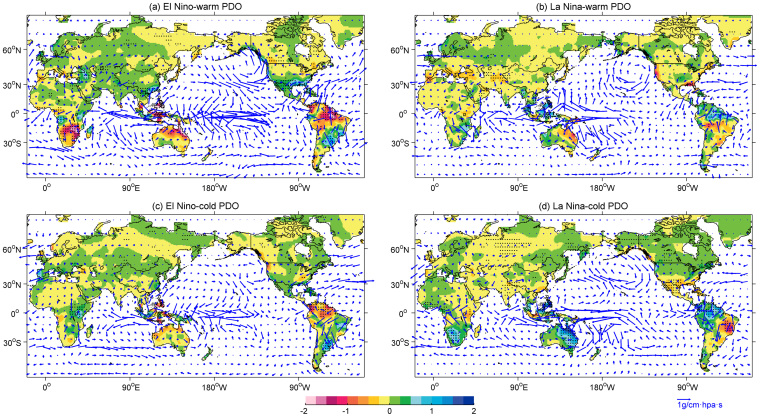
Composites of the DJF detrended 850 hPa water vapor flux (vector, g/cm·hpa·s) overlaid with PREC/L anomalies (shading, mm/day) for the period 1950–2012. (a) El Niño–warm PDO, (b) La Niña–warm PDO, (c) El Niño–cold PDO, and (d) La Niña–cold PDO. The stippling indicates a 90% confidence level according to a two-tailed Student's t-test. Maps and plots were produced using licensed Matlab.

An important question is why the ENSO-associated dry–wet anomaly distribution changes when combined with the different phases of the PDO. First, the underlying conditions — the combined SST distributions for the El Niño composite and the sub-composites according to the PDO phase in the boreal winter, are examined (Supplementary Fig. S7). The PDO and ENSO influence SST and circulation patterns in very similar ways, which is already found in the previous study^10^. The PDO primarily affects the North Pacific region and its effects can be felt near the equator. Conversely, ENSO primarily affects the lower latitudes, but its secondary effects are felt in the North Pacific^23^, as indicated in Supplementary Figs. S7a and S7d. When sub-composited by the PDO, the ENSO-related SST anomalies extend from the equatorial Pacific to the higher latitudes of the North Pacific via the eastern ocean during the in-phase condition, in addition to getting intensified over the equatorial Pacific. While El Niño (La Niña) occurs under the interdecadal-varying cold (warm) PDO background, the typical SST anomalies over the equatorial eastern Pacific get weakened and more concentrated along the equator; and the secondary signal over the North Pacific is much weaker even reverse. The corresponding atmospheric circulation at 500 hPa and water vapor transport at 850 hPa, including the geopotential height (HGT), the vertical velocity (omega), and the water vapor flux, are further examined (see Figs. 4 and 5). The El Niño signal over the North Pacific–North America region consists of anomalously low pressure around the Aleutian Islands and the southeastern US and opposite anomalies extending from the central Canada to Greenland, i.e., the positive Pacific/Northern America pattern (PNA). Further compositing with the warm PDO phase reveals that the HGT anomaly is largely similar to that depicted in Fig. 4a; however, the Aleutian Low is substantially stronger and a stronger high pressure center is concentrated over Canada (Fig. 4b). This enhanced positive PNA has been verified in previous studies^9^,^10^,^24^,^25^ and results in two branches of abnormally large amounts of water vapor over North America (Fig. 5a). One branch occurs along the southern flank of the Aleutian Low, transporting the water vapor from Pacific to the southwest, and the other branch brings the water vapor from Atlantic to the southeast. Therefore, more precipitation and magnified dry–wet changes are favored over North America (Fig. 1). In contrast, during the cold phase of the PDO, El Niño exhibits a similar while primarily insignificant HGT anomaly pattern over the North Pacific (weaker positive PNA, Fig. 4c) and less water vapor transport to the US (Fig. 5c), which results in relatively weaker aridity changes over North America. As for La Niña winters, especially those occurring during the cold PDO phase (Fig. 4f), the PNA is in its negative phase (Fig. 4d), while the water vapor transport is anomalously divergent around the southern US and Mexico (Fig. 5d).

Over the North Atlantic region, the negative North Atlantic Oscillation (NAO), which consists of an anomalously high pressure that extends over the eastern Canada, Greenland, and Iceland and anomalously low pressure that extends over the eastern US, the North Atlantic, and the Mediterranean region, is present during El Niño winters, especially during the cold PDO phase (Fig. 4c); this finding was previously mentioned by Gershunov and Barnett^10^. However, this NAO pattern is not noticeably present in combination with the warm PDO phase (Fig. 4b). As an important factor responsible for anomalous winters in Europe and the Mediterranean regions^26^,^27^,^28^, the negative NAO reduces the pressure gradient between the weak subtropical high and the weak Icelandic low and results in fewer and weaker winter storms crossing on a more west-east pathway; thus, moist air is transported into the Mediterranean region and cold air is transported into northern Europe (Fig. 5c). These changes induce moistening over the southern Europe and the tip of northwestern Africa and drying over the Scandinavian Peninsula (Fig. 1c). The opposite is true for La Niña winters; however, the effects are weaker, i.e., the positive NAO is relatively weak (Fig. 4e). Therefore, the corresponding dry–wet changes are insignificant over the Mediterranean region and Europe.

Moreover, there is a significantly abnormal high (low) pressure belt in the low latitudes during the El Niño (La Niña) winters, which is notably stronger and extends to eastern Asia during the in-phase condition. Thus, the El Niño-induced abnormal anti-cyclone over the western North Pacific basin is enhanced during the warm PDO phase, while it is weakened and retreats southeastward during the cold PDO phase. An enhanced anti-cyclone is always accompanied by a weaker than normal East Asia winter monsoon (EAWM)^29^, which results in a warmer and drier winter over the northern China. Meanwhile, the enhanced anomalous anti-cyclone transports an abundant supply of water vapor and favors much more precipitation than normal over southern China with respect to the cold PDO phase^30^. This modulation of the PDO on the ENSO-EAWM relationship has also been noted in previous studies^16^,^17^,^31^. The opposite is true for La Niña winters.

ENSO is also related to dry–wet variations over the Middle East, such as wetter (drier) conditions during El Niño (La Niña) winters over Irania^32^. When sub-composited by the warm PDO phase, El Niño-induced wetting is substantially intensified over the Middle East and central Asia, which abates the dry conditions over these arid regions. This effect is mainly contributed to the deepened trough of low pressure that extends from eastern Europe to the Middle East (Fig. 4b), which accelerates the Middle East jet stream and transports water vapor to the Middle East (Fig. 5a). When El Niño occurs during the cold PDO phase, these wetting conditions are relatively weak or reverse to the east of the Middle East. Correspondingly, La Niña-associated severe drying over the Middle East and central Asia is prominent during the cold PDO phase.

In addition, ENSO is the most dominant perturbation responsible for interannual climate variability over eastern and southern Africa^33^. Southern Africa is known to be affected by the ENSO with drier than normal conditions during El Niño and wetter conditions during La Niña due to the pressure anomalies over southern Africa and the Indian Ocean^34^. However, by comparing the composites of the sc_PDSI_pm under different conditions, this relationship is only stable and significant when ENSO and the PDO are in phase. When El Niño (La Niña) occurs with the warm (cold) PDO phase, the high (low) pressure anomalies over southern Africa and the Indian Ocean are stronger (Figs. 4b and 4f), thereby inhibiting (favoring) tropical convection and rainfall. This anomalous atmospheric circulation pattern is much weaker and more unstable in the out-of-phase condition. Unlike southern Africa, the typical rainfall anomaly over the Horn of Africa (eastern Africa) has a positive relationship with ENSO^33^. Specifically, the Horn of Africa dries during El Niño winters and becomes wetter during La Niña winters, as indicated in the present study. Further compositing by the PDO phase reveals additional details, when the PDO is in the cold phase, there is a robust relationship between ENSO and the dry–wet changes over the Horn of the Africa, especially since 1950 (Supplementary Fig. S5); however, the relationship is insignificant in the warm PDO phase.

Western Africa, including the Sahel, is inextricably linked to the West Africa monsoon circulation regime, which is responsible for most of the rainfall; however, its relationship with ENSO is more debatable^33^,^35^. As observed from the sc_PDSI_pm composites, a typical El Niño (La Niña) is associated with a slightly drier (wetter) belt over the Sahel. However, when further composited by the PDO, however, the Sahel to the Gulf of Guinea, especially to the east, experiences severe drying (wetting) when ENSO and the PDO are in phase, which is mostly due to the enhanced hot and dry Harmattan winds in the lower troposphere that do not occur during the out-of-phase condition.

In the tropical regions, the most prominent feature of the ENSO signal is the remarkable contrast in the vertical wind velocities associated with the Walker Circulation between the two phases of the PDO (Fig. 4, stippling). Note that the positive omega anomaly suggests enhanced descending motion or weakened ascending motion. Evidently, the ENSO-induced Walker Circulation anomaly is more prominently accompanied by stronger divergence/convergence of water vapor when in-phase with the PDO, but relatively weaker during the out-of-phase with the PDO (Fig. 5). Ascending motions associated with El Niño form Indonesia to Australia especially along the eastern coast, and northeastern South America, and descending motions over the central and northeastern Pacific weaken during the warm PDO phase relative to the cold PDO phase, which explains reasonably the dry–wet changes over these regions. For example, severe drying when El Niño occurs during the warm phase of the PDO occurs from Indonesia to Australia, especially to the east, and from northern Brazil to Colombia and Venezuela.

## Discussion

**Figure 6:**
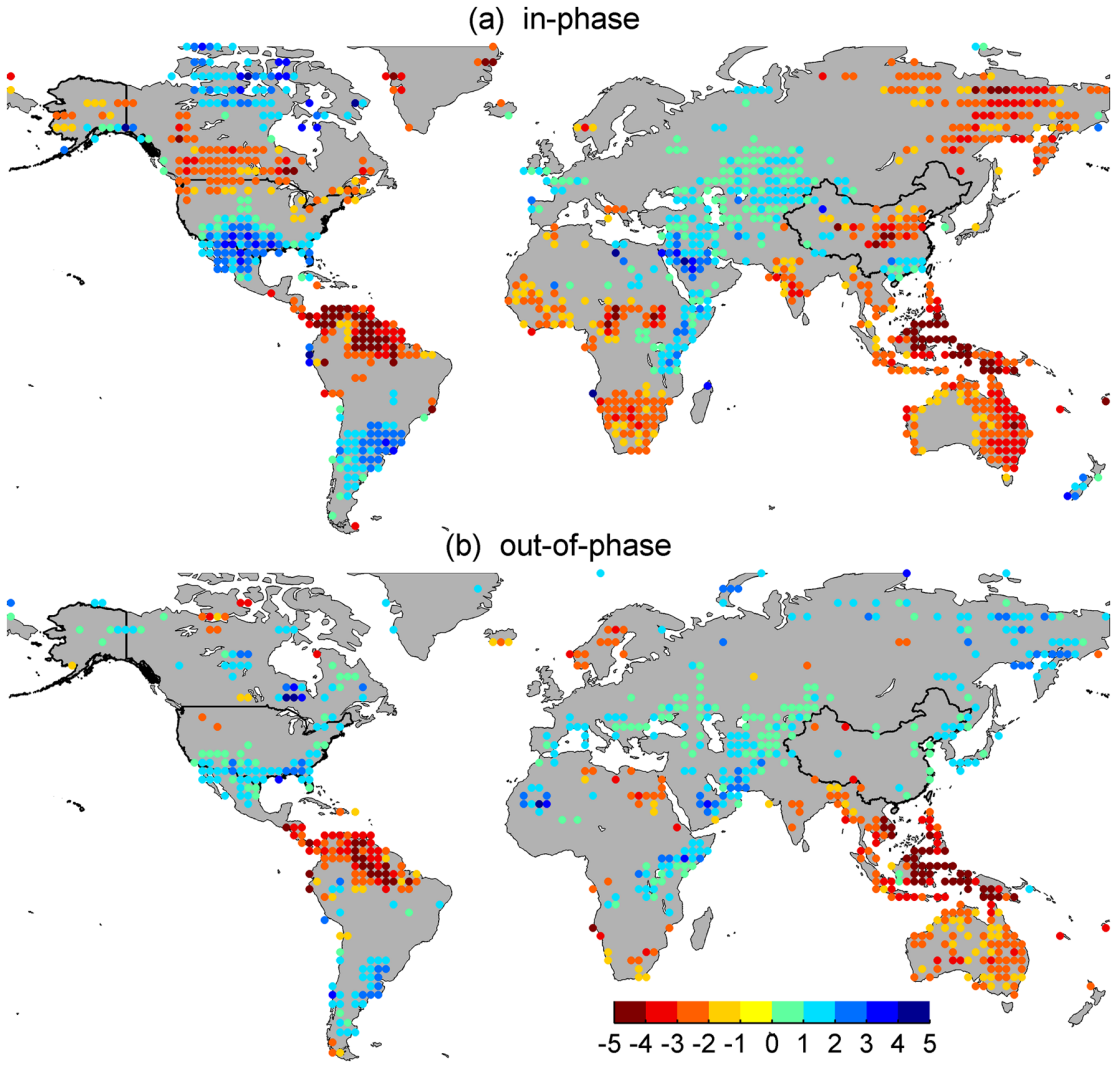
Differences in sc_PDSI_pm between El Niño and La Niña. (a) In-phase combination. (b) Out-of-phase combination. Colored circles indicate aridity changes greater than the 90% confidence level. Maps and plots were produced using licensed Matlab.

The ENSO-induced dry–wet distributions when combined with the PDO confirm the results obtained previously and reveal many important new features. When ENSO and PDO are in phase, the El Niño/La Niña-induced dry/wet anomalies are not only intensified over the canonical regions influenced by a typical ENSO event but also expand poleward (Fig. 6a). If ENSO and the PDO are out of phase, then the associated dry/wet anomaly is dampened or disappears (Fig. 6b). Generally, during the warm phase of the PDO, El Niño induces much broader and more severe droughts over land compared with the cold PDO phase. For example, the arid and semi-arid climate over the Sahel and southern Africa worsens. Correspondingly, during the cold phase of the PDO, more rain occurs over land in La Niña winters than during the warm PDO phase. However, there are a few exceptions. The amplitude of the dry–wet variation is larger over northern Europe and the Mediterranean during the out-of-phase condition, while the variation over the Horn of Africa tends to be stronger in the cold phase of the PDO.

The dynamics of the PDO remains very complex and climate models can't predict the future evolution of the PDO, especially the shift from one PDO phase to another. Even in the absence of a theoretical understanding, the PDO signal improves the climate forecasts combined with ENSO for different regions of the world due to its strong tendency for multi-decadal persistence. Around 2000, the PDO entered a cold phase with an increasing frequency of La Niña events, resulting in more rain over land^3^. Moreover, areas of La Niña-induced precipitation have become wetter and La Niña-induced drought areas have become drier; however, these areas are smaller due to the modulation by the cold PDO phase. For example, the contiguous US, especially the southwestern US, has experienced severe drought since the PDO moved into a cold phase around 2000 (US Drought Monitor^36^; http://www.drought.unl.edu/dm/monitor.html); many researchers have shown this phenomenon to be closely related to the tropical Pacific Ocean^3^,^18^,^37^,^38^. Moreover, the increased risk of ever-worsening drought in the US will persist in the coming decades, implying that the American drought is not likely to ease until the interdecadal PDO returns to a warm phase. Over the past decade, China has also experienced a dry–wet pattern turnaround — the pattern of “southern flood and northern drought” has become “southern drought and northern flood” — which seems likely to continue for another decade. Countries in southern Africa will experience rainfall and suffer more floods, and the semi-arid climate in the Sahel region will be abated. Central and southwest Asia will experience a persistent drought in the coming decades. In addition, floods and abnormally high rainfall are more likely in India, from the Indonesian islands to eastern Australia, and in equatorial South America. Meanwhile, southern Brazil and Uruguay have been prone to drought in recent decades. All of these effects of ENSO on global dry–wet changes as the interdecadal PDO enters a cold phase should be taken seriously in the coming decades, especially over the fragile dryland regions. Additionally, a new type of ENSO, ENSO Modoki, has occurred more frequently since the late 1970s^39^,^40^; however this event is not been discussed here due to the incomplete for the current PDO. This event should be considered in the future.

## Methods: Data

The drought index used here is the Dai's self-calibrated Palmer Drought Severity Index (PDSI) dataset (sc_PDSI_pm, http://www.cgd.ucar.edu/cas/adai/data-dai.html)^7^,^21^,^41^. In this dataset, potential evapotranspiration is calculated using the Penman–Monteith equation. The PDSI is calculated from a water-balance model that is forced by observed precipitation and temperature and is closely related to precipitation and soil moisture content; the PDSI has been widely used to study aridity changes. “*The revised sc_PDSI_pm has improved spatial comparability and uses a more realistic estimate of potential evapotranspiration, thus improving its applicability to global warming scenarios*^4^” Additionally, the widely used monthly precipitation for the period 1901–2012 from the CRU, the precipitation reconstruction over land (PREC/L) for the period 1950–2012 from the National Oceanic and Atmospheric Administration (NOAA)^42^, and the surface soil moisture (0 ~ 10 cm) for the period 1948–2010 from the GLDAS^22^ are also used here. The meteorological fields (i.e., geopotential height, u-wind, v-wind, omega, and specific humidity) are based on monthly mean National Center for Atmospheric Research first generation (NCEP1) reanalysis data for the period 1950–2012.

The PDO is defined as the leading EOF of the mean November–March sea surface temperature (SST) anomalies for the Pacific Ocean north of 20°N based on the HadSST 1900–1980, OI SST v1 1982–2001, and OI SST v2 2002–present datasets. For the EOF calculation, the global mean SST anomaly was first removed for each month to reduce the effects of the long-term trends in the data. Because the PDO is a largely interdecadal oscillation, an 11-year running average was performed to extract the decadal variability of the PDO. A warm (cold) PDO phase corresponds to above (below) zero values of the 11-year running mean PDO index.

ENSO extreme winters are defined to occur when the average December, January, and February (DJF) NIÑO34 detrended SST exceeds the long-term mean by 0.6°C. While high enough to exclude questionable events, this threshold provides an adequate number of ENSO cases when sub-composited with the PDO phase. The detrended DJF NIÑO34 SST was chosen as the ENSO index for two reasons: 1) it is well known that the NIÑO34 SST anomalies exert a strong effect on extratropical circulations during the boreal winter, and 2) the interannual NIÑO34 SST anomalies are most skillfully predicted by hybrid coupled models for DJF^10^. The classification of years associated with the phases of ENSO and the PDO for the period 1900–2012 is shown in Table 1. Note that in this study, winter is defined as the months of December, January and February (DJF). When performing the composite analysis, sc_PDSI_pm is detrended to remove the effects of global warming.

In this study, the stability of the ENSO signal is assessed via the cross-validated proportion of the intra-composite variance 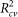 explained by the composite mean^9^. For each El Niño winter, the squared deviations from the mean of the remaining El Niño winters are computed at each grid for the sc_PDSI_pm. Then, the El Niño error sum of squares is calculated by summing this quantity over all El Niño winters: 
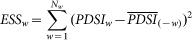
. Here, 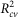 is calculated for all El Niño winters on each grid: 
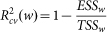
, where 
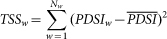
. Here, *w* indexes the El Niño winters, 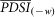 is the mean sc_PDSI_pm for all of the El Niño winters excluding winter *w*, and *TSS_w_* is the total sum of squares for the El Niño winters. Moreover, 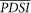 is the climatological mean of the sc_PDSI_pm for all winters. 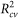 for La Niña is similarly computed from the La Niña winters. Due to the cross validation, negative values of skill are not only possible, but they are common in areas with little to no ENSO signal^9^. Only positive values of 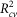 are contoured here.

## Author Contributions

All authors contributed to shaping up the ideas and writing the paper. J.H. and S.W. designed the study and contributed to data analysis, interpretation and paper writing. J.H., S.W., Y.H. and Y.G. contributed to the discussion and interpretation of the manuscript. S.W. and J.H. are co-first authors. All authors reviewed the manuscript.

## Supplementary Material

Supplementary Information Supplementary Information

## Figures and Tables

**Table 1:** Classification of years based on the phases of ENSO and the PDO for the period of 1900–2012. The year of 1900, for example, refers to the boreal 1900/1901 winter

	Warm PDO	Cold PDO
El Niño	1902,1904,1905,1911,1913,1923, 1925,1930,1939,1940,1941,1982, 1986,1987,1991,1994,1997,2002	1914,1918,1957,1963,1965,1968, 1972,1976,1977,2004,2006,2009
La Niña	1903,1908,1909,1910,1922,1924, 1933,1938,1942,1983,1984,1988, 1998,1999,2000	1916,1917,1949,1950,1954,1955, 1964,1970,1973,1975,2005,2007, 2008,2010,2011
