# Supplementary material for: Combined effects of the Pacific Decadal Oscillation and El Niño-Southern Oscillation on Global Land Dry–Wet Changes
Source: Sci Rep. 2014 Oct 17;4:6651. doi: 10.1038/srep06651 (PMC4200402; doi:10.1038/srep06651)
Supplement: Supplementary Information [file srep06651-s1.pdf]

Supplementary Information to

Combined effects of the Pacific Decadal Oscillation and El Niño-Southern Oscillation on Global Land Dry–Wet Changes

Shanshan Wang[1,2], Jianping Huang [1,2*], Yongli He [1,2], and Yuping Guan[3,4]

1. College of Atmospheric Sciences
Lanzhou University, Lanzhou, China

2. Key Laboratory for Semi-Arid Climate Change of the Ministry of Education
College of Atmospheric Sciences
Lanzhou University, Lanzhou, China

3. South China Sea Institute of Oceanology
Chinese Academy of Sciences, Guangzhou, China

4. State Key Laboratory of Tropical Oceanography
South China Sea Institute of Oceanology
Chinese Academy of Sciences, Guangzhou, China

Corresponding author: Dr. Jianping Huang
Key Laboratory for Semi-Arid Climate Change of the Ministry of Education
College of Atmospheric Sciences
Lanzhou University
Lanzhou 730000, China
hjp@lzu.edu.cn

FIGURE CAPTIONS FOR SUPPLEMENTAL MATERIALS:

Figure S1 | Composites of the JJA detrended sc_PDSI_pm during El Niño. (a) El Niño, (b) El Niño–warm PDO, and (c) El Niño–cold PDO for the period 1900–2012. (d) Differences between in- and out-of-phase with the PDO condition, i.e., (b) – (c). The stippling indicates a 90% confidence level according to a two-tailed Student's t-test. Maps and plots were produced using licensed Matlab.

Figure S2 | Same as in Fig. S1, but for La Niña. Maps and plots were produced using licensed Matlab.

Figure S3 | Composites of the DJF detrended CRU precipitation anomalies (mm/month) based on 1971–2000. (a) El Niño PDO, (b) La Niña PDO, (c) El Niño–warm PDO, (d) La Niña–warm PDO, (e) El Niño–cold PDO, and (f) La Niña–cold PDO for the period 1901–2012. The stippling indicates a 90% confidence level according to a two-tailed Student's t-test. Maps and plots were produced using licensed Matlab.

Figure S4 | Same as in Fig. S3, but for the DJF surface soil moisture anomalies (kg/m^2) for the period 1948–2010. Maps and plots were produced using licensed Matlab.

Figure S5 | Same as in Fig. S3, but for the DJF sc_PDSI_pm during the period 1950–2012. Maps and plots were produced using licensed Matlab.

Figure S6 | Same as in Fig. S3, but for the cross-validated proportion of variance (R_{cv}^2) in DJF sc_PDSI_pm during the period 1950–2012. Due to the cross validation, negative values of skill are not only possible, but common in areas with weak or no ENSO signal. Only positive values are contoured. Maps and plots were produced using licensed Matlab.

Figure S7 | Same as in Fig. S3, but for the DJF SST anomalies (℃) for the period of 1950–2012. Maps and plots were produced using licensed Matlab.

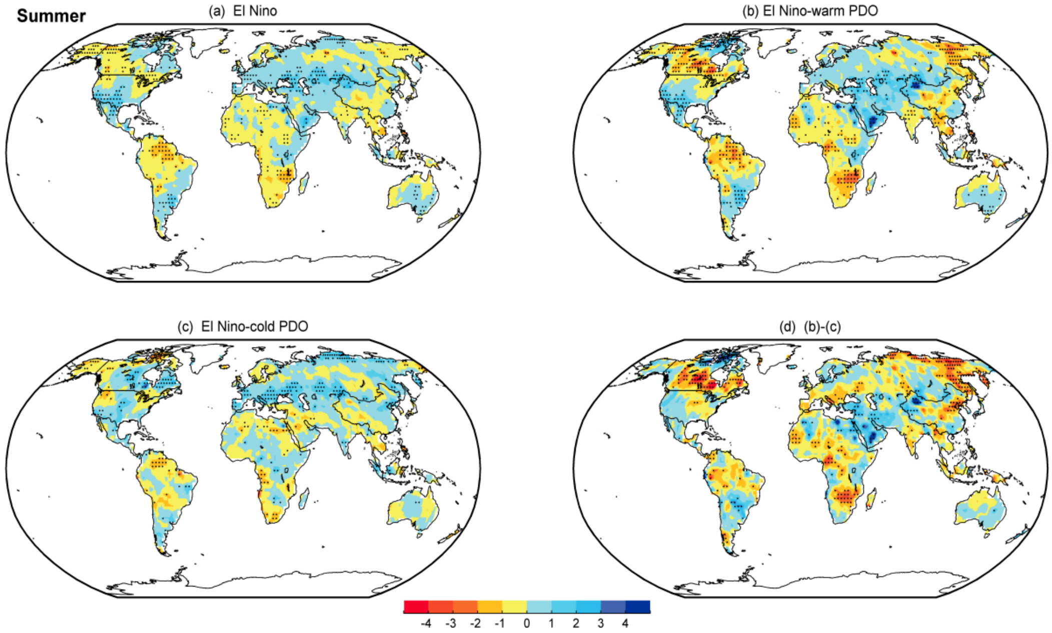

Figure S1 | Composites of the JJA detrended sc_PDSI_pm for El Niño. (a) El Niño, (b) El Niño–warm PDO, and (c) El Niño–cold PDO for the period 1900–2012. (d) Differences between in- and out-of-phase with the PDO conditions, i.e., (b) – (c). The stippling indicates a 90% confidence level according to a two-tailed Student's t-test. Maps and plots were produced using licensed Matlab.

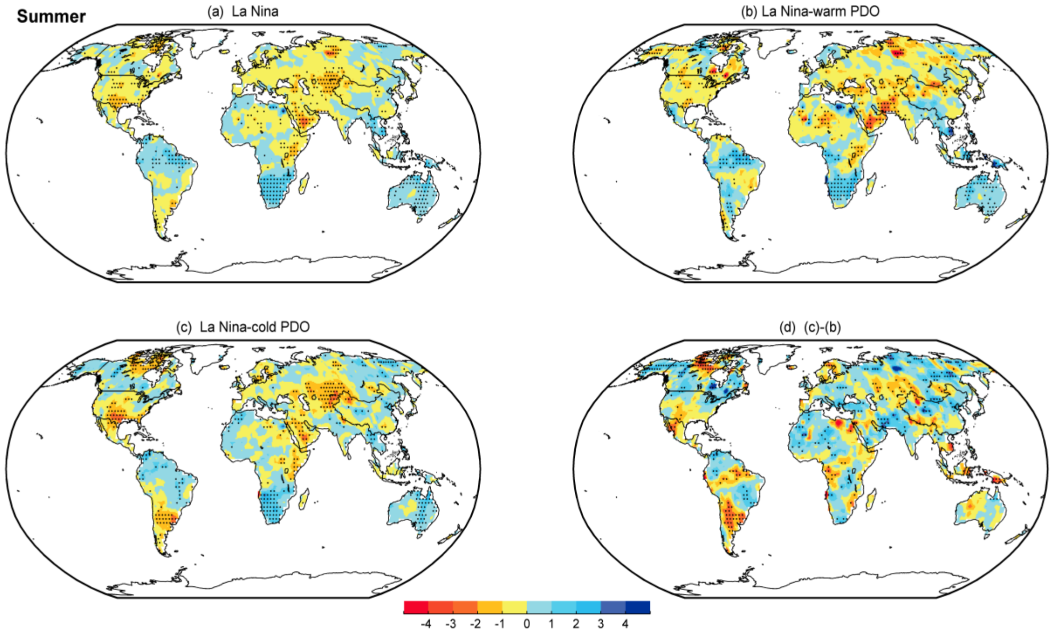

Figure S2 | Same as in Fig. S1, but for La Niña. Maps and plots were produced using licensed Matlab.

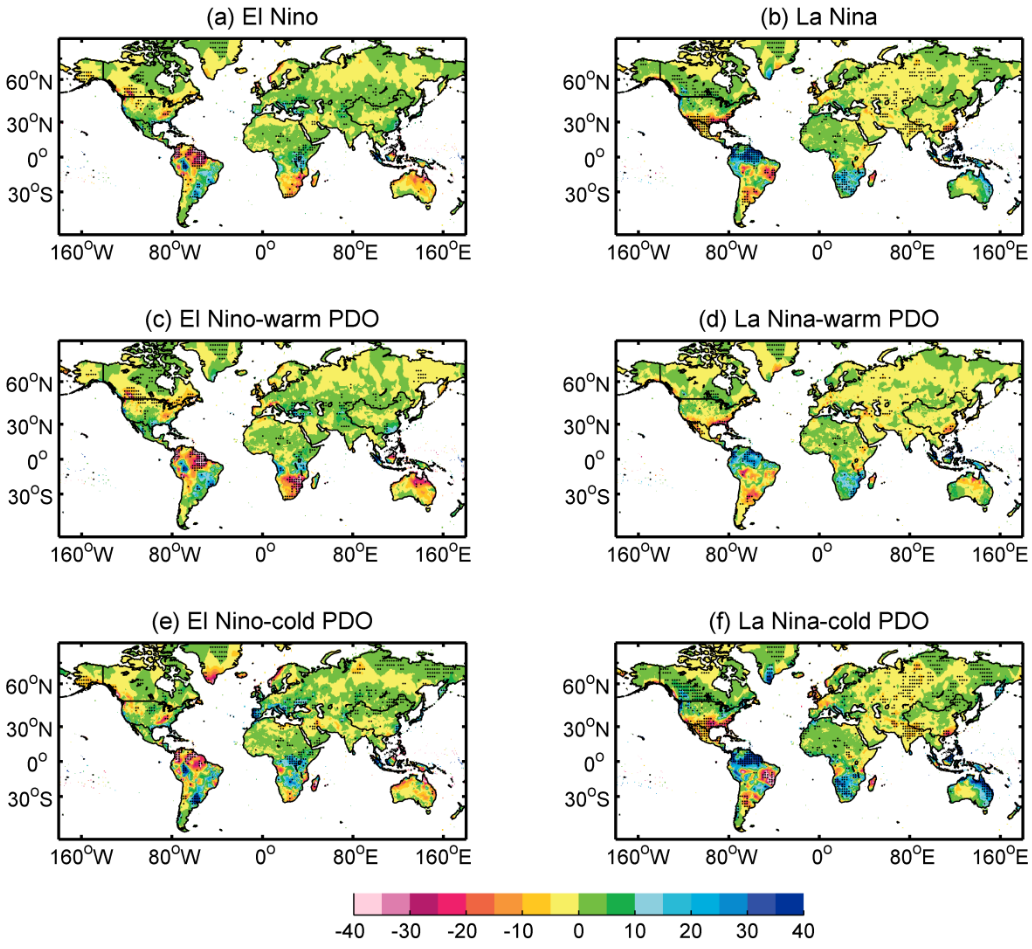

Figure S3 | Composites of the DJF CRU precipitation anomalies (mm/month) based on the period 1971–2000. (a) El Niño PDO, (b) La Niña PDO, (c) El Niño–warm PDO, (d) La Niña–warm PDO, (e) El Niño–cold PDO, and (f) La Niña–cold PDO for the period 1901–2012. The stippling indicates a 90% confidence level according to a two-tailed Student's t-test. Maps and plots were produced using licensed Matlab.

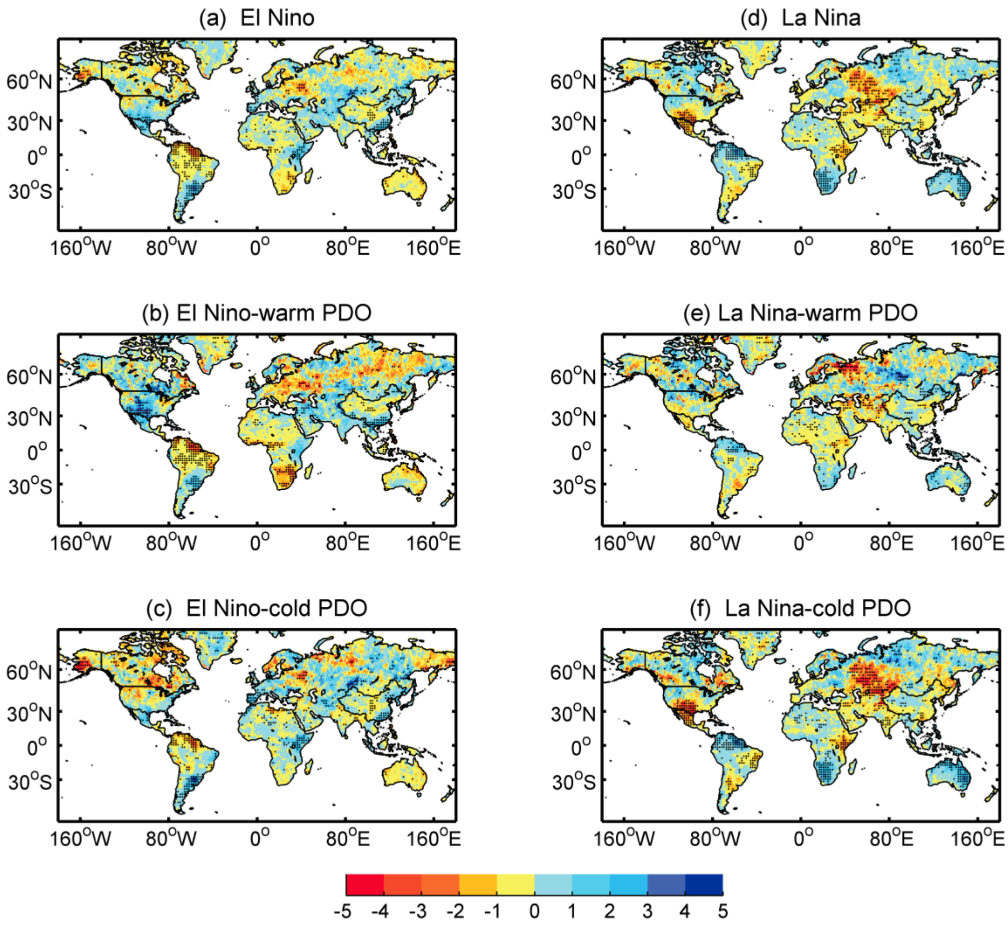

Figure S4 | Same as in Fig. S3, but for the DJF surface soil moisture anomalies (kg/m^2) for the period 1948–2010. Maps and plots were produced using licensed Matlab.

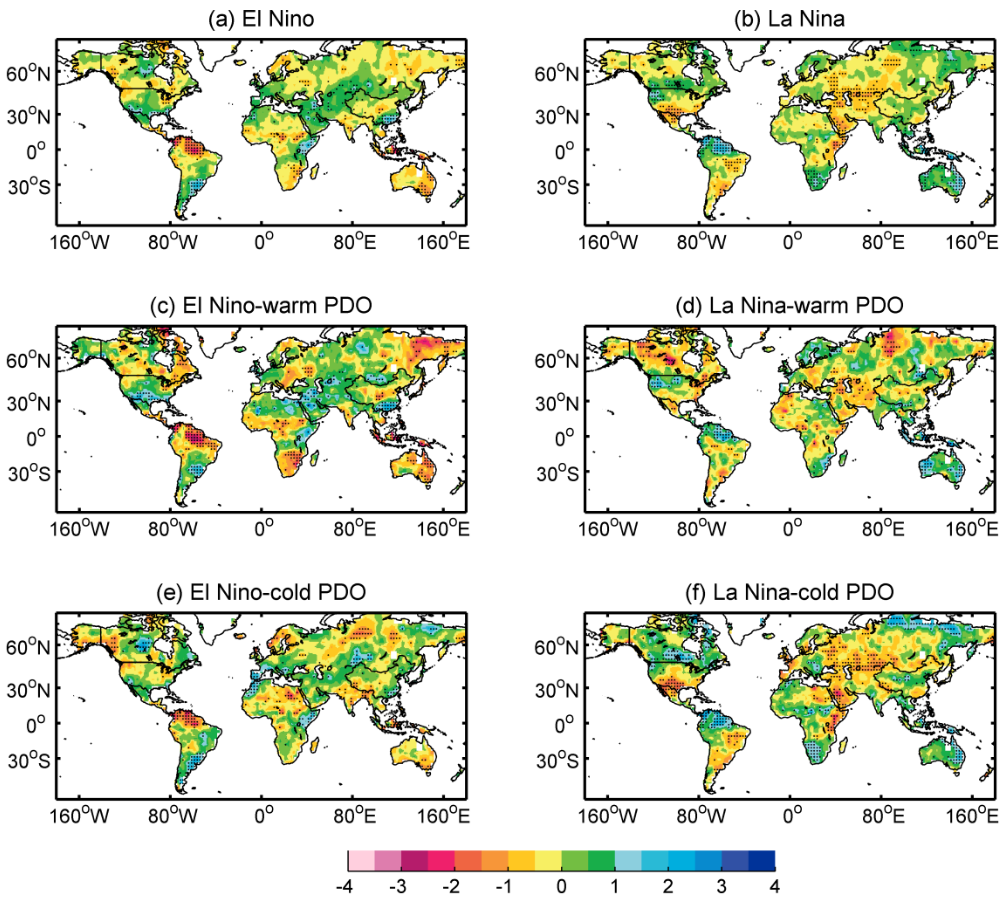

Figure S5 | Same as in Fig. S3, but for the DJF sc_PDSI_pm during the period 1950–2012. Maps and plots were produced using licensed Matlab.

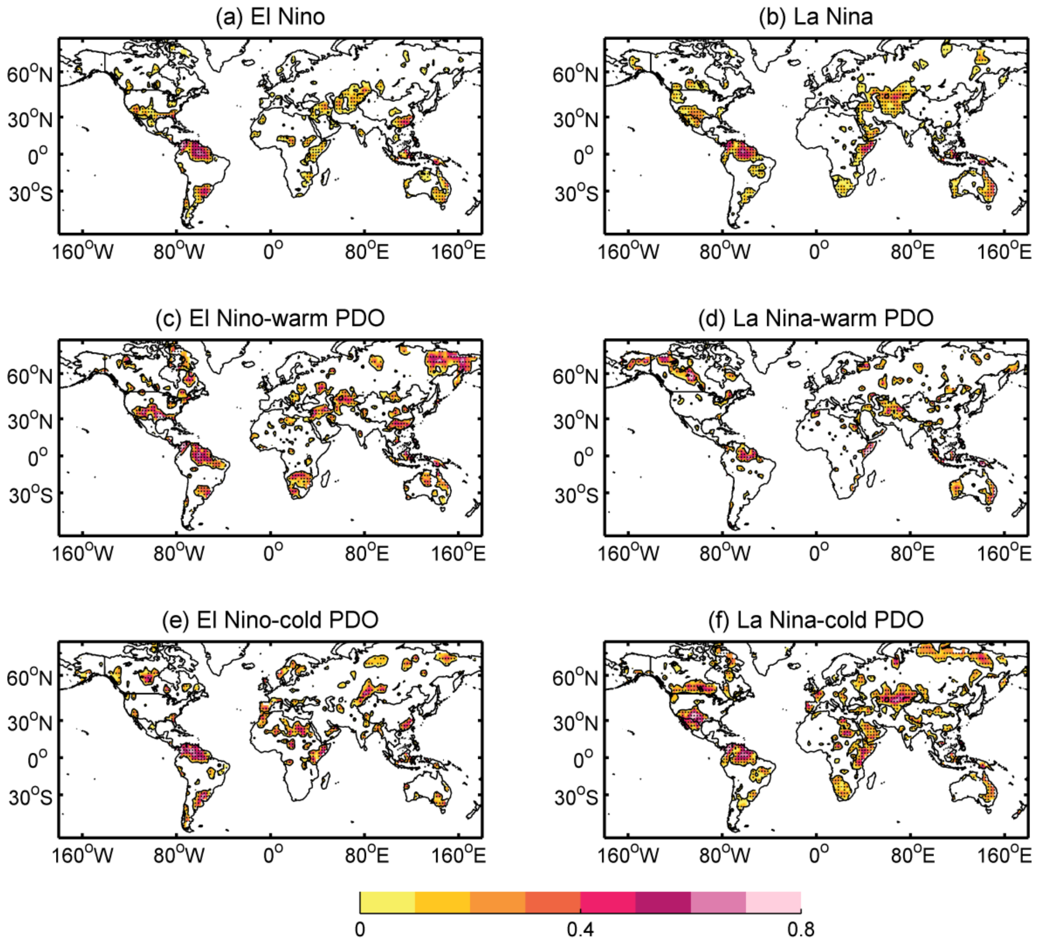

Figure S6 | Same as in Fig. S3, but for the cross-validated proportion of the variance (R_{cv}^2) in the DJF sc_PDSI_pm during the period 1950–2012. Due to the cross validation, negative values of skill are not only possible, but common in areas with no or weak ENSO signal. Only positive values are contoured. Maps and plots were produced using licensed Matlab.

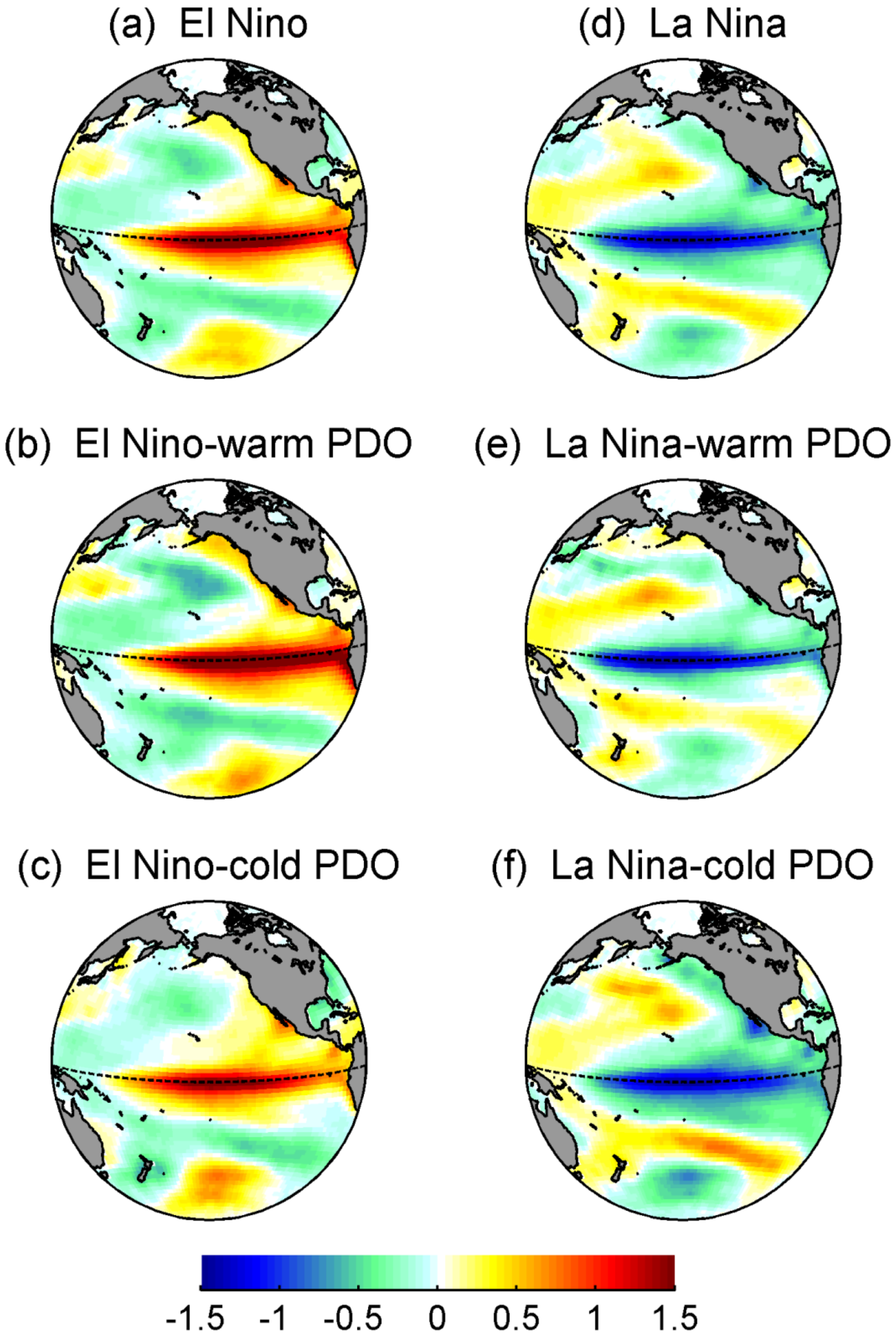

Figure S7 | Same as in Fig. S3, but for the DJF SST anomalies (℃) for the period of 1950–2012. Maps and plots were produced using licensed Matlab.
